# Periscope of Dermatology

**Published:** 1897-05-22

**Authors:** 


### Periscope of Dermatology.

(*Continued from page* 116).

**Pemphigus.**—Paul[9] describes a case of acute pemphigus which started with a violent dermititis, the skin over large areas of the trunk and extremities becoming red and raised, resembling erysipelas; on the surface of the inflamed areas bullæ were freely sprinkled. He also describes a case of a child, aged two months, who, after an attack of acute eczema of the face, developed bullæ all over him, which lasted for about a week.

Jon Jónsson[10] describes the case of a man of 50, in whom large bullæ extended over nearly the whole of the body. When these bullæ burst, more than two-fifths of the surface of the body was denuded of skin. The disease was accompanied by high fever; when this diminished, repair of the skin rapidly took place. No cause for the condition was found.

Duhring[11] considers the relationship of dermatitis herpetiformis to pemphigus, and considers the former condition a disease *sui generis*, which has tolerably constant distinct clinical features, and differs from pemphigus by the peculiar inflammatory and herpetiform character of the cutaneous lesions. He considers it more closely allied to erythema multiforme than to pemphigus.

Waldemar Peter[12] records a case of a child who developed acute pemphigus as a consequence of being fed with the milk of a woman suffering from septicæmia. The Demme diplococcus was found in the mother's milk, in the bullæ, and in the infant's blood.

Carrier[13] records two cases of dermatitis herpetiformis, and insists upon the distinctness of the disease from pemphigus. He noted that the lesions came in crops, and were accompanied by severe pruritis; these lesions gave place to erythema, which in turn was supplanted by vesicles or papules. He considers that these recurring attacks of the different lesions stamp the disease as dermatitis herpetiformis. For treatment, sulphur icthyol and a preparation of tar are the best local applications.

Manson[14] publishes a description by a medical man of his personal experience of dermatitis herpetiformis. He had suffered from the disease for over three years, and found that cocaine was the only thing that relieved the irritation; this he administered by spraying the nostril. He had tried every drug that had any reputation in the disease without success. The skin became better during attacks of remittent fever, and during an attack of diarrhœa, but relapsed again when these diseases were cured. He found the itching more intense when no vesicles were present on the skin.

Pringle[15] reports the case of a cab driver who developed an attack of dermatitis herpetiformis, commencing on the back of the neck. Eosinophile cells were found in the blood. A deep abscess formed on the left hand, after which large bullæ developed all over the body, with septicæmic symptoms. After the septicæmia had lasted over five weeks, the dermatitis herpetiformis was cured. It, however, relapsed some months later.

Whitfield[16] found, on examining the blood in a case of dermatitis herpetiformis, that coarse multinuclear eosinophiles formed 25 per cent. of all the leucocytes present. The serum from bullæ also showed large numbers of the same eosinophiles, and these were in greater numbers than any other leucocytes. The blood was stained with Ehrlick's triple stain.

**Sclerodermia.**—Dreschfeld[17] records two cases of diffuse sclerodermia, in one of which marked muscular atrophy, and in the other trophic ulcers were present.

Officer[18] records the case of a child, aged 18 months, who within the course of a few days developed sclerodermia over nearly the whole of the body and limbs. It started on one cheek, and the next day the other cheek was noticed to be red and hard. Within one week the whole body and limbs were affected. The mucous membrane of the mouth was also affected. The case improved under thyroid feeding.

Dercum[19] relates three cases of sclerodermia, in one of which the disease followed an injury to the back three months previously, and so he attributed the disease to the nervous shock of the accident. The second case was marked by the universal disappearance of the subcutaneous fat, and the involvement of the phalangeal joints, as shown by Röntgen's rays. Pains suggesting neuritis were complained of, and trophic ulcers occurred over the phalangeal joints. In the third case there were pronounced atrophic changes on the skin of the face, forearms, and hands, together with ankylosis of the finger joints.

West[20] relates a case of localised sclerodermia in a young woman, in which the skin lesion occurred in two long streaks, about two to three inches wide, along the inside of the left thigh, and on the front of the left leg, corresponding to the distribution of the third lumbar segment. No neuralgic pain accompanied the lesions.

**Acne.**—Abrahams[21] recommends subcutaneous injections of a 5 per cent. alcohol solution in cases of acne rosacea; 20 to 30 drops are injected into the diseased part about three times a week till a cure is effected. The immediate effect of the injection is swelling and anæmia of the parts; this lasts but a few moments, and is followed by increased redness which lasts from half to three or four hours. The dilated blood vessels and papules will be found, after repeated injections, to undergo slow but sure obliteration, until finally the whole lesion disappears. The treatment lasts from eight to ten weeks.

Betz[22] has found the embrocation of turpentine of use in the treatment of acne rosacea. It causes at first violent smarting and redness which soon disappear. He suggests that the drug has a solvent action on the sebaceous secretion, besides acting as a disinfectant and producing hyperæmia.

Lomry[23] has investigated the etiology of acne from the bacteriological side. He finds that the staphylococcus albus is present in almost every pustule, but this is little virulent unless passed through animals. Other micro-organisms are found in the pustules, but the skin of people without acne is as rich in microbes as that of those with it. He cultivated Unna's bacillus, and states that it is in reality a little virulent variety of the bacillus coli commune.

[9] Lancet, Feb. 15, 1897, p. 437. [10] Ditto, March 15, 1897, p. 736. [11] Am. Journ. of Med. Sci., Feb. 1897, p. 169. [12] Brit. Journ. of Derm., Feb., 1897, p. 84. [13] Med. Rec., Jan. 2, 1897, p. 9. [14] Brit. Journ. of Derm., March, 1897, p. 97. [15] Ditto, Dec., 1896, p. 484. [16] Ditto, Jan., 1897, p. 36. [17] Med. Chron., Jan., 1897, p. 263. [18] Aust. Med. Gaz., Dec. 21. 1897, p. 517. [19] Brit. Med. Journ., Ep., Dec. 26, 1896. [20] Brit. Journ. of Derm., Nov. 1896, p. 443. [21] Ther. Gaz., Sept. 15, 1896. p. 618. [22] Lancet, Jan. 30, 1897. [23] Brit. Journ. of Derm., Nov., 1896, p. 456.

